# Structural stress response of segmented natural shells: a numerical case study on the clypeasteroid echinoid *Echinocyamus pusillus*

**DOI:** 10.1098/rsif.2018.0164

**Published:** 2018-06-13

**Authors:** Tobias B. Grun, Malte von Scheven, Manfred Bischoff, James H. Nebelsick

**Affiliations:** 1 Department of Geosciences, University of Tübingen, Hölderlinstraße 12, 72074 Tübingen, Germany; 2 Institute for Structural Mechanics, University of Stuttgart, Pfaffenwaldring 7, 70569 Stuttgart, Germany

**Keywords:** structural mechanics, skeleton, computed tomography, finite-element analysis, voxel-based, load-carrying behaviour

## Abstract

The skeleton of *Echinocyamus pusillus* is considered as an exceptional model organism for structural strength and skeletal integrity within the echinoids as demonstrated by the absence of supportive collagenous fibres between single plates and the high preservation potential of their skeletons. The structural principles behind this remarkably stable, multi-plated, light-weight construction remain hardly explored. In this study, high-resolution X-ray micro-computed tomography, finite-element analysis and physical crushing tests are used to examine the structural mechanisms of this echinoid's skeleton. The virtual model of *E. pusillus* shows that the material is heterogeneously distributed with high material accumulations in the internal buttress system and at the plate boundaries. Finite-element analysis indicates that the heterogeneous material distribution has no effect on the skeleton's strength. This numerical approach also demonstrates that the internal buttress system is of high significance for the overall skeletal stability of this flattened echinoid. Results of the finite-element analyses with respect to the buttress importance were evaluated by physical crushing tests. These uniaxial compression experiments support the results of the simulation analysis. Additionally, the crushing tests demonstrate that organic tissues do not significantly contribute to the skeletal stability. The strength of the echinoid shell, hence, predominantly relies on the structural design.

## 1. Introduction

Sea urchins (Echinodermata, Echinoidea) have increasingly become the focus of structural analyses as their light-weight skeletons feature a remarkable load-carrying property (e.g. [1–16]). The echinoid skeleton is a hierarchically organized (e.g. [9]) and multi-plated construction that functions as a protective shell for internal organs. The light-weight skeletons are built from a lattice-like stereom with a porosity of up to 50% [5]. The plates of most echinoid skeletons are securely interconnected by collagenous fibres (e.g. [2–4]), which often disarticulate quickly after soft tissues decay. The clypeasteroid echinoids feature additional strengthening mechanisms that enhance skeletal integrity: in addition to the collagenous fibres, these echinoids developed skeletal protrusions bridging the sutures between plates (e.g. [17–20]). The skeletons of this echinoid group are common and often well-preserved in both recent environments and the fossil sedimentary record [11,21–28], which is indicative for a structurally resilient skeletal architecture [20]. The minute *Echinocyamus pusillus* represents a particular case within the clypeasteroid echinoids as collagenous fibres between the plates are entirely absent [3].

**Figure 1.**
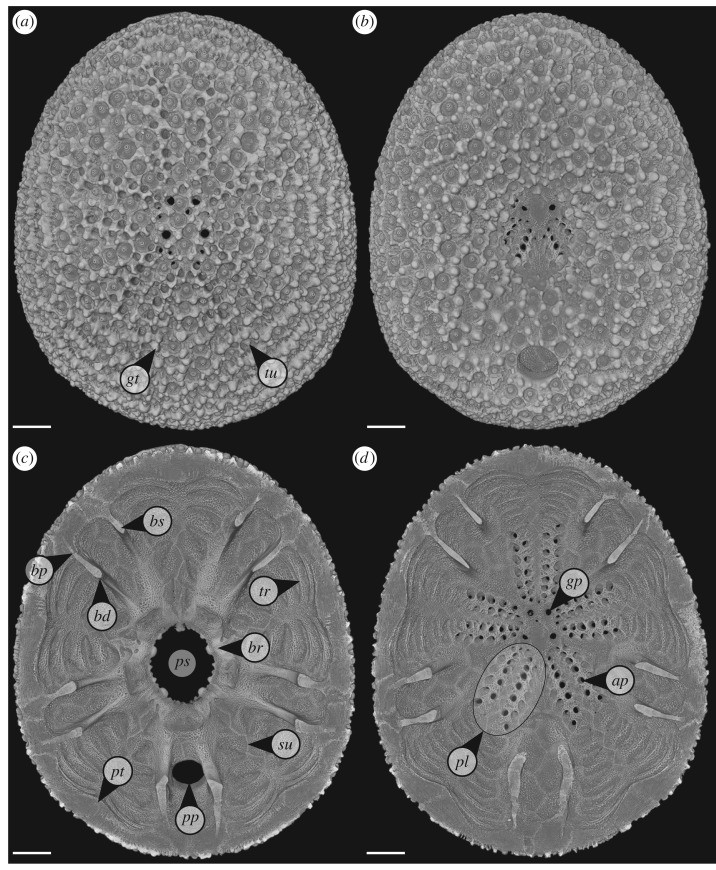
Micro CT rendering of *E. pusillus* from Giglio island, Italy. (*a*) Skeleton in aboral view. The ambulacral system and microstructures such as the tubercles and glassy tubercles are visible. (*b*) Skeleton in oral view with the prominent peristome and periproct visible. (*c*) Top view of the oral side. The internal supports are visible, as well as the basicoronal ring around the peristome. (*d*) View on the inner part of the aboral side. Pores and the limits of the buttresses are visible. *ap*, ambulacral pore; *bd*, distal region of the buttress; *bp*, proximal area of the buttress; *br,* basicoronal ring; *bs*, buttress; *gp,* genital pore; *gt,* glassy tubercle; *pl,* petal; *ps*, peristome; *pt*, plate thickening; *pp,* periproct; *su,* suture; *tr,* transversal ridge; *tu,* tubercle. Scale bar, 500 µm.

*Echinocyamus pusillus* feature four structural strengthening mechanisms, the buttress system, plate thickening at the sutures, transversal ridges and the previously mentioned plate interlocks [3,19]. The internal buttress system consists of five pairs of radially wall-like buttresses which extends from the interambulacral basicoronal plates towards the outer margins of the petals (e.g. [3,10,21,28–35]) (figure 1). Buttresses and plate thickening have been interpreted to strengthen the skeleton when loads are applied by bridging the oral and aboral side of the echinoid [3,10,21,28,30,35,36]. Furthermore, the traversal ridges of the ambulacral plates which range from the peristome to the outer limits of the petals are thought to increase the flexural stiffness of the skeleton (e.g. [3]). Along with the internal supports, this clypeasteroid features tight-fitting and interdigitating plate joints (e.g. [3,18,19,36]). These internal reinforcements and plate interlocking render the *E. pusillus* skeleton resistant to destruction (e.g. [3,10,18,19,21,28,35]).

Despite these findings, there have to date been few if any analytical or numerical studies concerning the density distribution and structural parameters important for the strengthening mechanisms of the clypeasteroid skeleton. The identification and analysis of structural strengthening mechanisms based on the echinoid skeleton are crucial for developing new techniques in building constructions of segmented shells. Reciprocally, understanding the basis for enhanced skeletal integrity can be useful in reconstructing both fossilization pathways and the role of these clypeasteroids in ancient ecosystems. This study, accordingly, investigates structural aspects of the morphology of the clypeasteroid echinoid *E. pusillus* with respect to the significance of the internal support systems and further strengthening mechanisms. High-resolution three-dimensional (3D) X-ray micro-computed tomography (µCT) is used to obtain a digital model of the echinoid's skeleton. The 3D model is used as the basis for material density mapping and a finite-element model. Physical crushing tests were additionally conducted to examine the structural importance of the internal buttresses and organic materials.

### 1.1. The role model *Echinocyamus pusillus*

The minute irregular echinoid *E. pusillus* features a flattened and subcircular skeleton (figure 1), which rarely exceeds a length of 20 mm. With the skeleton entirely covered by minute spines (e.g. [37]), this infaunally living echinoid burrows through soft substrates using both spines and tube-feet [38]. *Echinocyamus pusillus* inhabits a wide range of environments including muddy [39], silty [40], sandy [28,37,38,41], shell-gravel [38,41] as well as poorly sorted sediments [42]. In coarse sandy environments, the echinoid lives between gravel particles, masking itself by carrying sand grains on its aboral side (e.g. [38,42]). The spines have been shown to absorb up to 20% of occurring loads [3].

The abundance of well-preserved skeletons from both fossil and recent environments, as well as the initial structural analyses of *E. pusillus* (e.g. [3]) demonstrates that the skeleton of this echinoid can be considered as a valuable role model for biomimetic research on segmented shells.

### 1.2. Previous finite-element analyses on echinoids

The complex skeletal geometry and the varying thicknesses of the skeleton limited structural simulations and thus the understanding of biomechanical principles of the echinoid skeleton in the past. Only few finite-element analyses have been conducted on simplified and abstracted echinoid skeleton models [5,6]; hence, the understanding of form and its mechanical function is poorly known, although the high skeletal variability of these organisms has been described in detail (e.g. [43]). Initial studies on the stress distribution have been performed on a simplified computer-aided geometric design model of regular echinoids [5,6]. Such a model allows for parametrization of predetermined characters by keeping computational time low. The often closely spherical regular echinoids feature a pentamerous symmetry that allows a subdividing of the entire skeleton into five nearly identical units. Analyses of a single unit can then be interpreted on the entire skeleton without losing information as the skeleton is made from five of these segments (e.g. [5]). The pivotal analysis of Philippi & Nachtigall [5] and Philippi [6] indicated that loads can be effectively carried by the double-curved design, though loading was not crucial for the echinoid shape. It was assumed that tensile stress due to the tube-feet required for locomotion is a significant driver for the specific test form.

In *E. pusillus*, methods and results employed by Philippi & Nachtigall [5] and Philippi [6] cannot be applied: the skeleton of this clypeasteroid follows an overprinted bilateral symmetry resulting in a skeleton that cannot be reduced to a number of similar segments. Additionally, *E. pusillus* lives buried within the sediment where it uses spines for burrowing, resulting in various skeletal adaptations not found in regular echinoids. As high-resolution imaging techniques and computational resources have increased over the past decades together with the development of advanced finite-element methods, a much more detailed investigation of echinoid skeletons can be applied. In this study, the skeleton of *E. pusillus* is analysed for the first time for its mechanical performance using an accurate 3D model based on µCT scans.

## 2. Material and methods

### 2.1. Material

Denuded skeletons of *E. pusillus* were collected in summer 2010 during scuba dives around the island of Giglio (Tyrrhenian Sea, Italy: 42°21'07.9″ N 10°52'52.1″ E) and from beach sediments at Riccione (Adriatic Sea, Italy: 44°01'17.9″ N 12°38'13.6″ E) in September 2014. Additional specimens conserved in alcohol (70% vol.) were obtained from the Alfred-Wegener-Institut, Helmholtz-Zentrum für Polar- und Meeresforschung, Helgoland, Germany, collected in the North Sea. Samples are stored at the Department of Geosciences, University of Tübingen, Germany, under repository GPIT/EC/00740 for Giglio and GPIT/EC/00756 for Helgoland. Coordinates are obtained from Google Maps 2017.

### 2.2. Three-dimensional models

An X-ray µCT scan of *E. pusillus* (specimen GPIT/EC/00740:gg-al-1.73) was obtained by a Phoenix Nanotom (General Electric Company Corporation, Boston, MA, USA) at the German Aerospace Center (Deutsches Zentrum für Luft- und Raumfahrt), Stuttgart, Germany. The scan was performed with an isotropic voxel size of 3 µm, and scanning parameters as follows: voltage = 80 kV, power = 180 µA, exposure time = 800 ms, projections = 2000. The µCT 3D scan was recorded to an 8-bit grey-scale system with 878 image slices in the *X*–*Y* (horizontal) plane in JPG file format and a resolution of 1934 × 2320 pixels.

**Figure 2.**
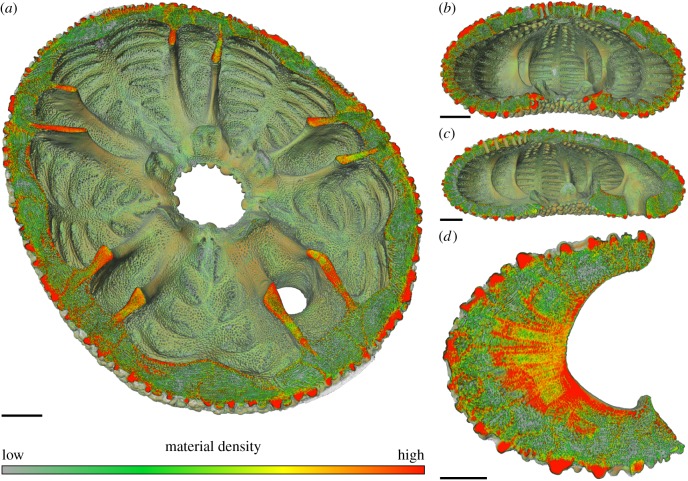
Colour-coded 3D rendering of *E. pusillus*. (*a*) Section showing the material distribution within the plates and the internal supports in horizontal and oblique view. Red indicates high material densities at the outer surface (tubercles and glassy tubercles), sutures and within the buttress system. (*b*) Frontal section of the skeleton. Plate centres are dominated by a low material density (green). (*c*) The lateral section shows that material densities are similarly distributed along the longitudinal axis. (*d*) A detailed section of a single buttress shows that the material density is highest (red) in direction to the peristome and along the suture areas. The average stereom of the buttress is dominated by intermediate dense (yellow) material. Scale bar, 500 µm.

The µCT imagery was rendered in FEI Avizo in v. 9.4.0 (Thermo Fisher Scientific, Waltham, MA, USA) with active grey values ranging between 42 and 255 for the final model. The grey-scaled model was transferred into a colour-coded model by re-assigning threshold grey values to a RGB colour space (figure 2 and table 1).

**Table 1.** Conversion values from grey-scale to RGB colour space applied to the active grey values range between 42 and 255.

grey	colour (R, G, B)
42.00	255, 255, 255
95.25	170, 170, 170
148.50	0, 214, 42
201.75	254, 255, 0
255.00	255, 24, 0

### 2.3. Voxel finite-element model

**Figure 3.**
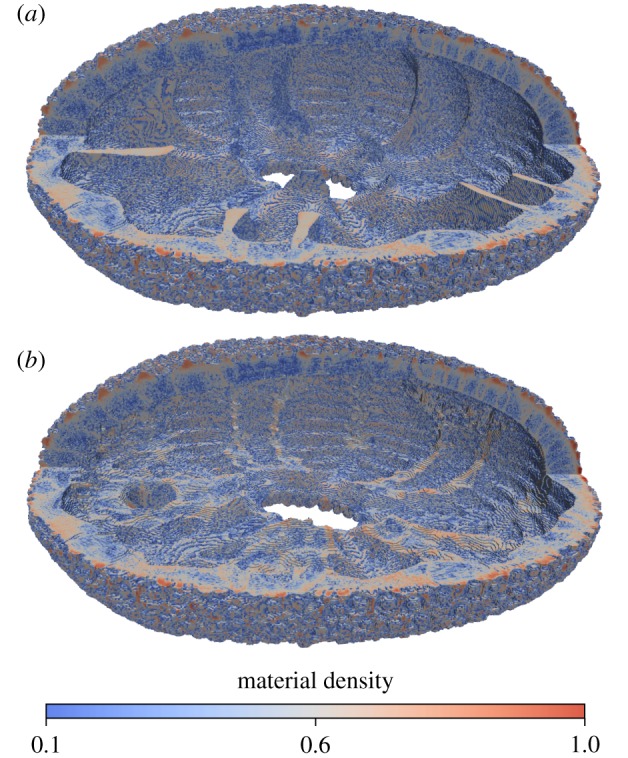
Voxel model of *E. pusillus*. (*a*) Original geometry including the buttresses and heterogeneous material distribution. (*b*) Modified geometry without the buttresses and heterogeneous material distribution.

Based on the µCT imaginary, a finite-element model [44–46] for the structural analysis of an *E. pusillus* specimen was generated. This process was automated in the numerical computing environment Matlab (MathWorks, Natick, USA) and consists of the voxel-based reconstruction [47–49] of the three-dimensional geometry including information about the density, removal of floating parts of material and the generation of different loads and boundary conditions. To reduce the computational effort for the finite-element simulation, 4 × 4 × 4 pixels are combined to produce one voxel element with averaged material properties, represented by the grey values of each pixel. The resulting model (figure 3*a*) consists of 7 927 311 voxels, 10 450 890 nodes and 31 230 685 degrees of freedom.

Philippi & Nachtigall [5] performed tests to obtain material properties for the regular echinoid *Echinus esculentus*. Their value for the Young's modulus of non-perforated interambulacral areas of *E* = 9.50 kN mm^−2^ is used here as a base value. Poisson's ratio was assumed to be *ν* = 0.2, a value characterizing brittle biomaterials. The organic material was not considered for the virtual tests as physical tests have shown that organic tissues do not affect the mechanical behaviour of the skeleton.

To include the non-homogeneous material distribution in the finite-element analysis, the grey values from µCT scans were linearly related to the density of the material. For the dependence of Young's modulus on the density or porosity of the material, a nonlinear relation [16,50] is used,
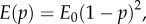
with *E*_0_ denoting the base value for the Young's modulus and *p* the porosity of the material.

The finite-element analysis of the voxel model is performed using our in-house research code ‘NumPro' taking advantage of the regular voxel structure for computational efficiency [51,52]. The matrix–vector product used in the preconditioned conjugate-gradient method, used for equation solving, is implemented in a matrix-free way [53] using pre-calculated products on voxel level. Furthermore, the implementation is parallelized by openMP using a shared memory approach.

### 2.4. Virtual tests

To understand the structural load-carrying behaviour of the skeleton of *E. pusillus*, virtual experiments are conducted. Hereby, variations of the geometry and model parameters can be examined that are not available for real physical tests. To investigate the significance of the material distribution in the structure and the buttress system for load-carrying behaviour, four different test cases are examined:

A. Original geometry including the buttresses; heterogeneous material distribution
B. Original geometry including the buttresses; homogeneous material distribution
C. Modified geometry without the buttresses; heterogeneous material distribution
D. Modified geometry without the buttresses; homogeneous material distribution

In cases C and D the buttresses are removed during generation of the voxel model, resulting in an approximately constant skeleton thickness (figure 3*b*). For the homogeneous material distribution, the same amount of material of the original model is distributed homogeneously to keep the total mass constant.

Loads and boundary conditions are defined similar to the physical crushing tests. A ring-shaped area on the lower side of the skeleton is supported in vertical direction and a circular area on the upper side is loaded by a constant pressure with a resultant force of 1.6 N in vertical direction. A linear static analysis is sufficient to obtain the stress distribution in the skeleton and determine load-carrying characteristics.

### 2.5. Physical crushing tests

The physical crushing tests are performed to evaluate results obtained by finite-analysis methods. Additionally, these uniaxial compression experiments are used to identify the role of organic tissues around the stereom. Therefore, skeletons of *E. pusillus* (GPIT/EC/00756) were analysed for the force needed until the skeletal structures fail. Three treatment groups are compared to identify the effect of organic material in the skeleton and the presence of the internal buttress system. Samples of all treatment groups possess all appendages and tissues, and are stored in ethanol (70%). Skeletons are prepared by gently milling the aboral side down to a third of the skeleton's height. This procedure ensures that forces are applied on both the ambitus and the internal supports directly. Treatment group 1 includes skeletons, where the jaw apparatus and organs of the skeleton cavity were removed by tweezers. In treatment group 2 and treatment group 3, all organic material was removed in a 60 min bath in a 12% solution of sodium hypochlorite after it was washed under running tap water. The buttress system of treatment group 3 was removed using pointed tweezers.

The uniaxial compression tests were performed using a PCE-FB dynamometer (PCE Instruments, Meschede, Germany). For these experiments, samples are placed on an aluminium sample stub (Plano GmbH, Wetzlar, Germany) mounted on a height-adjustable stage. During elevation of the stage, samples are pressed against a plate stamp of the measuring device. The elevation stops when the skeleton is crushed.

A Kruskal–Wallis *H* test is used to compare skeleton lengths among treatment groups to ensure that the three treatment groups are similar in size. The resulting forces for skeletal failure along the treatment groups are compared by a Kruskal–Wallis *H* test followed by a pairwise Benjamini, Hochberg and Yekutieli *p*-adjusted Wilcoxon *post hoc* analyses to identify possible effects of the organic material and internal supports with respect to the skeletal strength. The comparative crushing analysis conducted in the present study does not aim to provide absolute numbers for the skeleton's load-bearing capacity, but compares the original skeleton morphology of *E. pusillus* to manipulated structures with the goal of recognizing the importance of organic tissues and internal buttressing for skeletal strength.

Measurements are reported in newtons (N) using the median for the measure of central tendency, and the median absolute deviation (mad) for the measure of dispersion. A major axis regression is performed employing the R package ‘smatr' [54,55] analysing the relation between the force loading capacity of the skeleton and the skeleton's length.

## 3. Results

### 3.1. Material distribution

The colour-coded 3D model of *E. pusillus* (figure 2) shows the material density distribution of the structural elements. The outer surface of the skeletons is of high material density (red). These structures can be identified as tubercles and glassy tubercles that mostly consist of imperforated stereom. In horizontal section (figure 2*a*), plates mostly consist of low (green) or intermediate (yellow) material density. In the sutural areas where plates are interconnected to one another, the density is higher (yellow to red).

In frontal section (figure 2*b*), the outer surface of plates where tubercles and glassy tubercles are present show a high material density in contrast to the plate's inner areas which typically show lower material densities. The inner transversal ridges show higher material densities along with that of plate boundaries. Material densities are similarly distributed over the entire width of the skeleton. In lateral section (figure 2*c*), material densities are similarly distributed over the longitudinal axis.

The material density found in the buttress system shows a generally higher stereom density than in the remaining skeleton (figure 2*a*). Areas of high material density are thereby mainly accumulated in the buttress areas towards the peristome as shown in the section of an individual buttress (figure 2*d*). Sutural areas are of high material density whereas most of the central buttress is dominated by intermediate-to-high material density. The modified skeletons, where internal supports have been virtually removed, show an approximately constant skeletal thickness (figure 3*b*).

### 3.2. Virtual tests

**Figure 4.**
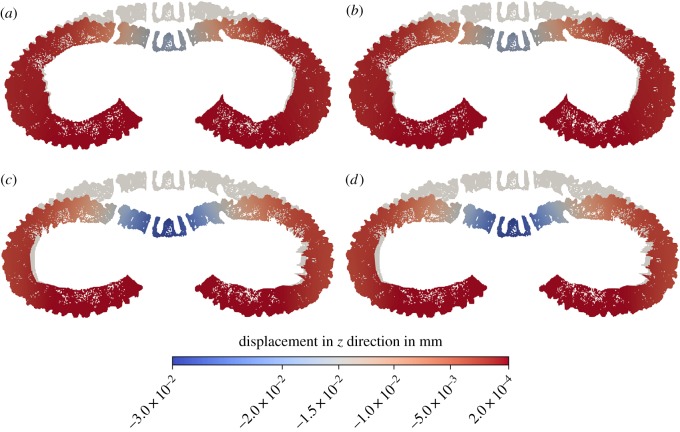
Undeformed (grey) and deformed (coloured) section of the voxel model of *E. pusillus.* Deformation is scaled by a factor of 25. The colours show the magnitude of the vertical displacement. (*a*) Original geometry including the buttresses with heterogeneous material distribution. (*b*) Original geometry including the buttresses with homogeneous material distribution. (*c*) Modified geometry without the buttresses with heterogeneous material distribution. (*d*) Modified geometry without the buttresses with homogeneous material distribution.

The vertical displacement caused by applied loadings is compared for the four test cases (A–D) of the model. The displacements for the original skeleton geometry with the buttress system present and heterogeneous material distribution (case A, figure 4*a*) and with the buttress system present and homogeneous material distribution (case B, figure 4*b*) are practically identical. By contrast, the displacement for the modified geometry without the buttresses are around 57% larger for both heterogeneous and homogeneous models (cases C and D, figure 4*c,d*) than for the original geometry.

**Figure 5.**
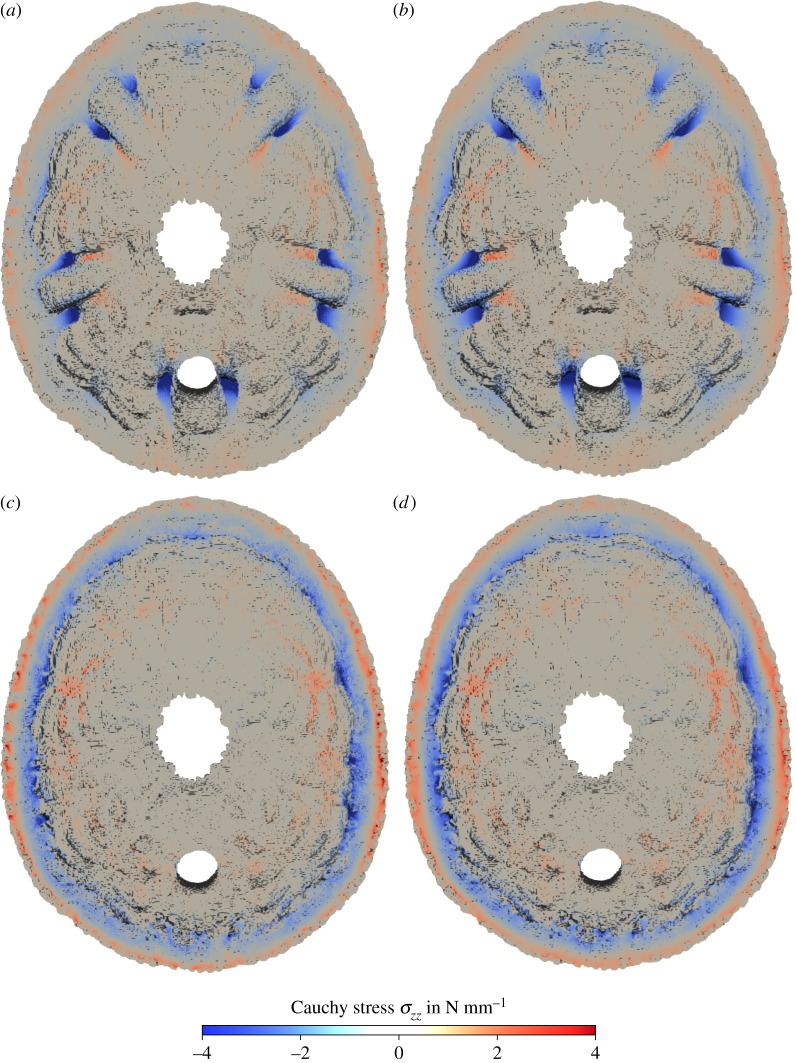
Contour plot of the Cauchy stresses in *z* direction in a section of the voxel model of *E. pusillus.* (*a*) Original geometry including the buttresses with heterogeneous material distribution. (*b*) Original geometry including the buttresses with homogeneous material distribution. (*c*) Modified geometry without the buttresses with heterogeneous material distribution. (*d*) Modified geometry without the buttresses with homogeneous material distribution.

**Figure 6.**
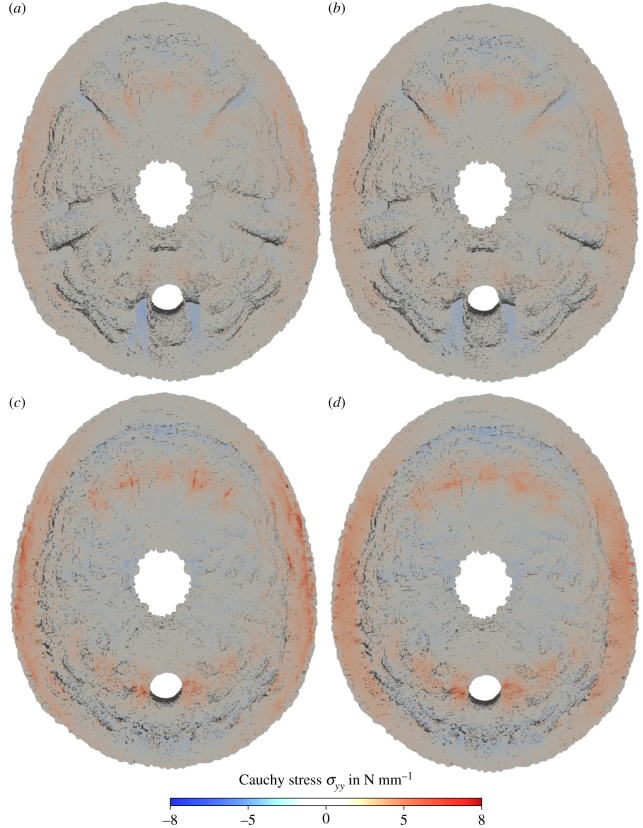
Contour plot of the Cauchy stresses in *y* direction in a section of the voxel model of *E. pusillus*. (*a*) Original geometry including the buttresses with heterogeneous material distribution. (*b*) Original geometry including the buttresses with homogeneous material distribution. (*c*) Modified geometry without the buttresses with heterogeneous material distribution. (*d*) Modified geometry without the buttresses with homogeneous material distribution.

In the comparison of the distribution of normal stresses in vertical direction *σ*_zz_ in horizontal section of the original skeleton of *E. pusillus*, it can be observed that the maximum compressive stress occurs in the buttress areas towards the peristome for both the heterogeneous and homogeneous model (figure 5*a*,*b*). For the models without buttresses, larger bending stresses with high tension on the outer surface are visible in the finite-element graph (figure 5*c*,*d*). The tensile stress in circumferential direction (stress *σ*_yy_) in the upper and lower part of the horizontal section is also larger for the modified geometry without the buttresses (figure 6).

### 3.3. Physical crushing tests

**Figure 7.**
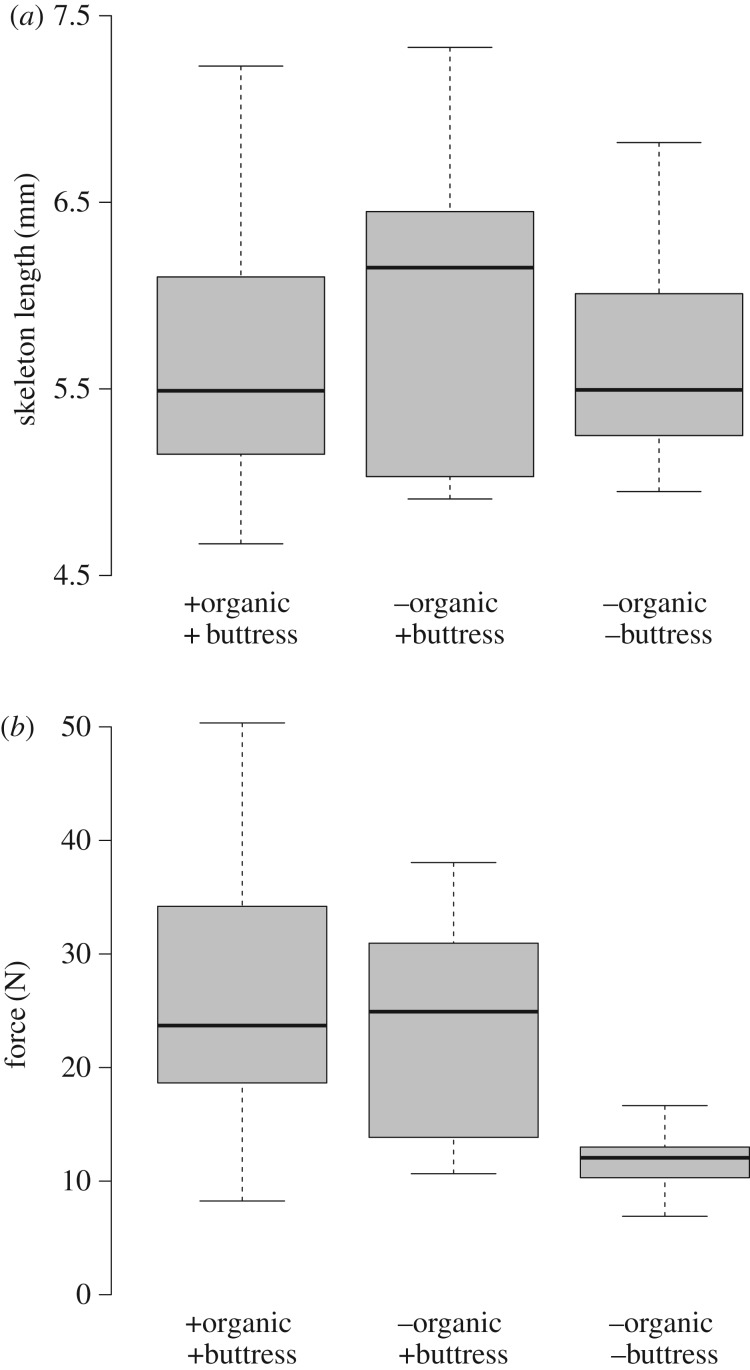
Box plots of statistical analyses for the load-bearing capacity of the skeleton along three treatment groups. (*a*) Skeleton length comparison indicating that the analysed skeletons are of similar size classes along the treatment groups. (*b*) The comparison of force applied until structural failure occurs indicates that buttressing is a structurally importantly parameter.

A Kruskal–Wallis *H* test compared 30 *E. pusillus* skeletons indicating that the three treatment groups do not statistically differ in their average skeleton length (*χ*^2^ = 1.29, *p* = 0.526, *N* = 30) (figure 7*a*). A Kruskal–Wallis *H* test followed by a pairwise *post hoc* analysis shows that the force needed until the skeleton crushes does not statistically differ between the group with organic material with internal supports present and the group where organic material was removed, but internal supports are present (*p* = 1.000, *N* = 20). The group with organic material and internal supports present maintain higher force loadings than skeletons where both organic material and internal supports were removed (*p* = 0.006, *N* = 20). Similarly, the test group without organic material, but with internal supports present, shows a higher force load capacity than the test group where both the organic material and internal supports were removed (*p* = 0.025, *N* = 20).

The test group where organic material and the internal supports were present shows an average force load capacity of 23.70 N (figure 7*b* and table 2). The test group where organic material was removed, but internal supports are still present, shows an average force load capacity of 24.93 N. The test group where both the organic material and the internal supports were removed shows an average force load capacity of 12.05 N.

**Table 2.** Measurements of *E. pusillus* skeleton length and forces needed until structural failure.

treatment	group 1	group 2	group 3
	+organic +buttresses	−organic +buttresses	−organic −buttresses
skeleton length (mm)			
median	5.49	6.15	5.50
mad	0.83	1.10	0.45
min.	4.67	4.91	4.95
max.	7.23	7.33	6.82
force (N)			
median	23.70	24.93	12.05
mad	8.52	13.31	2.48
min.	8.25	10.65	6.90
max.	50.35	38.05	16.65
*N*	10	10	10

## 4. Discussion

### 4.1. Material distribution

The skeleton of *E. pusillus* possesses a high variation in material distribution (figure 2). Variations in material distribution of the stereom reflect both mechanical and functional aspects. The outer surface of the skeleton is covered by tubercles for spine attachment and glassy tubercles of high material density. The distribution of tubercles and glassy tubercles has been discussed to increase skeletal integrity, as these microstructures can cross plate boundaries [4]. The material distribution within the skeletal plates shows notable accumulations on the plate sutures, where plates are connected to one another (figure 2). These areas are recognizable as yellow to red zones in the colour-coded images indicating that interdigitating plates [3,18,19] locally increase the material density. This plate interlocking results in a monolithic shell behaviour and is thus important for the structural integrity of the skeleton [18,19]. The highest material accumulation can be found in the buttress system. These structures have also been shown to transfer most of the applied stress (figure 5).

The approximately constant skeleton thickness of the modified model (figure 3) is a relevant criterion for the finite-element analyses, as highly varying thicknesses can affect the stress distribution and hence the results. The voxel renderings show that the areas, where buttress elements have been removed, are in the range of thickness of the original skeleton geometry.

### 4.2. Mechanical load-bearing behaviour

The investigation of the four models of the virtual test does not provide any information about the strength of the skeleton of *E. pusillus* in its natural environment, but the results can be used to understand the load-bearing behaviour of the skeleton. Furthermore, by comparison of the heterogeneous and homogeneous models, reasons for the material distribution can be deduced.

The vertical displacement caused by the applied loading is directly related to the overall stiffness of the skeleton of *E. pusillus.* By comparing the displacements for the four test cases of the virtual tests, the influence of different parameters on the stiffness can be evaluated. The displacements for the original skeleton geometry with heterogeneous (A) and homogeneous (B) material distribution are practically identical (figure 4*a*,*b*). This indicates that the qualitative load-bearing behaviour of the skeleton, in particular the stress distribution, is practically independent of the density distribution. The stiffness of the skeleton mostly depends on the overall geometry. It is a structural property rather than a material property. The material distribution is not optimized for the stiffness of the overall load-bearing behaviour. Still, the strength of the material and the overall structure depend on the heterogeneous density distribution.

The importance of the geometry and the buttresses for the overall stiffness can also be observed by comparing the displacements of the original geometry (figure 4*a*,*b*) and the modified geometry (figure 4*c*,*d*). Without the buttresses, the displacements are around 57% larger for the heterogeneous and the homogeneous models than for the original geometry.

The maximum compressive normal stress in vertical direction *σ*_zz_ in a horizontal section through the original skeleton of *E. pusillus* (figure 5*a*,*b*) occurs in the buttress areas towards the peristome for both the heterogeneous and homogeneous models. In structural mechanics, usually stiffer material attracts forces. For *E. pusillus*, however, the stress concentration in the buttresses is not caused by the higher density in these areas, as it can also be observed for the homogeneous model. The stress distribution is rather due to the geometry and, therefore, a structural property. Consequently, the higher density of the material in the buttress areas towards the peristome is caused by the high stresses to obtain a higher strength (as opposed to stiffness) of the material.

The positive effect of the buttresses on the load-bearing behaviour is obvious in the comparison of the stress distribution: for the model without buttresses, larger bending stresses with high tension are present on the outside of the skeleton (figure 5*c*,*d*). This bending is caused by the local loading but is also due to the fact that for this thick shell the ratio between membrane stiffness and bending stiffness is not as large as for thin shells. The tensile stress in circumferential direction (stress *σ*_yy_ in the upper and lower part of the horizontal section) is also much larger without the buttresses (figure 6). If the vertical load is carried by the pure shell of the modified geometry, large tension in circumferential direction is needed for equilibrium. The buttresses cause a load-bearing behaviour more similar to a rib vault resulting in much lower tension in circumferential direction.

### 4.3. Physical crushing tests

The uniaxial compression tests show that internal buttressing is relevant for the skeletal stability (figure 7). This result supports the outcome of the finite-element analysis which supports the interpretation that the internal supports do in fact strengthen the skeleton of *E. pusillus*. A structural strengthening behaviour has often been attributed to the buttressing structures (e.g. [3,4,17,28,56]), yet these assumptions have been tested neither physically, nor virtually. This is, however, not surprising as the naturally grown shells vary in geometry, thickness, curvature and porosity, parameters which are crucial for property determination (e.g. [57]).

Although it has been previously shown that collagenous fibres are absent within the plates' sutures [3], the presence of soft tissues such as the epidermis which covers the entire skeleton, or the stroma within the stereom interspace [58], have not been analysed in this context. Results of the physical crushing tests show that organic tissues do not contribute to a significant increase in skeletal strength (figure 7). This is an important result, as it demonstrates that the load-bearing capacity of this echinoid's skeleton predominantly relies on the structural design, rather than on supportive tissues. This fact makes the skeleton of *E. pusillus* an attractive role model for structural engineering, where segmented shells become increasingly important (e.g. [10,11]).

## 5. Conclusion

(1) Material distribution in the skeleton of *E. pusillus* is heterogeneous with high material accumulations in the sutures and the buttress system (figures 2 and 3).
(2) Finite-element analyses show that models of the skeletons with heterogeneous material distribution have a similar overall stiffness compared to those models with homogeneous material distribution (figures 4–6). This result thus implies that the geometry of the skeleton is more important for the overall stiffness than the material distribution.
(3) Finite-element analyses furthermore show that internal buttressing is crucial for load transfer and minimizing bending and tension of the skeleton. Removing the buttresses causes large bending stresses in meridian direction and high tensile circumferential forces around the peristome (figure 6).
(4) Physical crushing tests support that the results of finite-element analysis are plausible. The uniaxial compression experiments also indicate that organic tissues have no significant effect on the skeletal strength (figure 7); thus the structural integrity of the skeleton of *E. pusillus* predominantly relies on its skeletal design.

## Data accessibility

The µCT data of *Echinocyamus pusillus* are accessible via Dryad Digital Repository under http://dx.doi.org/10.5061/dryad.rg54h [18,19].
